# Learning Hyperbolic Embedding for Phylogenetic Tree Placement and Updates

**DOI:** 10.3390/biology11091256

**Published:** 2022-08-24

**Authors:** Yueyu Jiang, Puoya Tabaghi, Siavash Mirarab

**Affiliations:** 1 Electrical and Computer Engineering, University of California San Diego, La Jolla, CA 92093, USA; 2 Halıcıoğlu Data Science Institute, University of California San Diego, La Jolla, CA 92093, USA

**Keywords:** distance-based phylogenetics, phylogenetic placement, gene sequence embedding, deep learning, metric tree embedding, hyperbolic spaces

## Abstract

**Simple Summary:**

We show how the conventional (Euclidean) deep learning methods developed for phylogenetics can benefit from using hyperbolic geometry. The results point to lowered distance distortion and better accuracy in updating trees but not necessarily for phylogenetic placement.

**Abstract:**

Phylogenetic placement, used widely in ecological analyses, seeks to add a new species to an existing tree. A deep learning approach was previously proposed to estimate the distance between query and backbone species by building a map from gene sequences to a high-dimensional space that preserves species tree distances. They then use a distance-based placement method to place the queries on that species tree. In this paper, we examine the appropriate geometry for faithfully representing tree distances while embedding gene sequences. Theory predicts that hyperbolic spaces should provide a drastic reduction in distance distortion compared to the conventional Euclidean space. Nevertheless, hyperbolic embedding imposes its own unique challenges related to arithmetic operations, exponentially-growing functions, and limited bit precision, and we address these challenges. Our results confirm that hyperbolic embeddings have substantially lower distance errors than Euclidean space. However, these better-estimated distances do not always lead to better phylogenetic placement. We then show that the deep learning framework can be used not just to place on a backbone tree but to update it to obtain a fully resolved tree. With our hyperbolic embedding framework, species trees can be updated remarkably accurately with only a handful of genes.

## 1. Introduction

Phylogenetic trees capture the evolutionary history of species and, more importantly, define a space in which we can compute *distances* between biological entities. Considered in this light, a phylogeny *T* is a discrete metric space in which path lengths provide a proper distance between species. The distance between every two species, vertex pairs in *T*, depends on the topology of *T* and its edge lengths. We may embed a phylogeny, or a metric tree in general, by representing it in a low-dimensional continuous space while preserving certain properties of the tree [1]. Embedding provides a vector representation for the tree vertices, enabling continuous optimization algorithms for downstream learning tasks.

As de Vienne et al. [2] point out and Layer and Rhodes [3] further elaborate, for a tree *T* with *N* vertices, there exists an embedding of its vertices in $R^{N-1}$ such that Euclidean distances of the embeddings match with the square root of the corresponding tree distances. Embedding phylogenetic trees in Euclidean spaces opens the possibility of adopting rich toolkits developed for studying objects in Euclidean spaces. For example, Phylo-MCOA [2] is based on principal component analysis applied to embedded trees, and it finds outlier species in specific genes in a phylogenomic dataset.

Jiang et al. [4] use the insight from Layer and Rhodes [3] to adopt deep learning methods for phylogenetic placement: the problem of adding a set of novel *query* sequences onto an existing *backbone* tree. Euclidean embedding of the backbone species enables them to pose phylogenetic placement as a supervised learning problem. They show that one can learn a map between the sequence and Euclidean spaces while best preserving the metric information of the backbone tree. Their proposed method, DEPP, uses neural networks to map DNA sequences to a high-dimensional Euclidean space while matching squared distances of the embedded points with the tree distances. The neural network embeds new query sequences (aligned to the backbone) in the same space, which then enables us to compute their distances to the backbone species. Finally, we can use distance-based phylogenetic placement methods such as APPLES [5,6] to place the queries onto the tree.

Why would we use machine learning to compute distances when we could instead directly compute distances from sequences? In the application explored by Jiang et al. [4], the measurements are single-gene sequences that evolve on the gene tree, but the backbone is the species tree. Therefore, the main challenge is the discordance between the gene sequences and the backbone species trees. The goal of DEPP is to allow one to extend a species tree given limited data coming from a single or a handful of genes. Note that when researchers use marker genes such as 16S, they are interested in species relationships, not gene relationships, and for them, gene tree discordance is a nuisance. While updating a species tree using a single gene may seem an ill-posed objective, the biological application is real. In particular, species trees with tens of thousands of microbial species [7,8] have been put together with much computational effort and using hundreds of marker genes. Such trees can be used as the backbone for the placement of microbial samples.

Low-distortion tree embedding is the key concept in the phylogenetic placement approach proposed by Jiang et al. [4]. By embedding the tree in a Euclidean space, they relax the combinatorial nature of working with trees and sidestep the difficulties of training a model parameterized in a discrete space. After this relaxation, in the continuous space, they define a simple differentiable cost function and minimize it using standard back-propagation and stochastic gradient descent techniques. While the use of Euclidean geometry has some justifications, it is not clear that a fixed-dimensional Euclidean space is the best choice for embedding trees. In fact, Euclidean geometry (with its zero curvature) has severe limitations in terms of the number of dimensions needed to obtain low-distortion embeddings, as we elaborate in Section 2.2.

A natural alternative to Euclidean space is a non-Euclidean space with constant nonzero curvature. In recent years, there has been a growing appreciation that hyperbolic spaces are more suitable than Euclidean spaces for representing tree-like data; refer to Section 2.3. These realizations have motivated machine learning researchers to propose methods to represent *pairwise* measurement extracted from a tree in hyperbolic spaces [9,10,11]. For example, hyperbolic neural networks seek to combine the power of hyperbolic geometry with the feature learning capabilities of neural networks [12,13]. A major goal of the present work is to take advantage of these recent developments for phylogenetic placement. That being said, utilizing hyperbolic geometry comes with its own set of practical challenges. One issue is the negative impact of limited bit precision in representing embeddings [14]. Another major challenge in using hyperbolic geometry is performing arithmetic operations using hyperbolic addition and multiplication. Various authors have approached these issues in different ways [12,13,15]. The present work wrestles with many of the same difficulties; for a detailed discussion, refer to Section 2.4.3.

**Figure 1:**
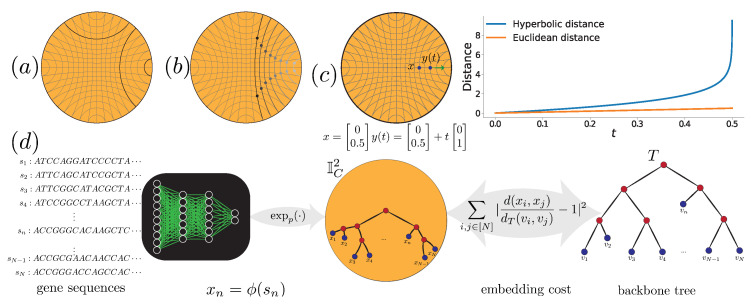
(**a**) Lines (or geodesics) in hyperbolic space. Bold lines are parallel to each other. (**b**) From left to right, the hyperbolic distance between the same-colored points remains constant as they get closer to the boundary of the space. (**c**) Hyperbolic distances grow unboundedly as points move towards the boundary. (**d**) The framework of H-DEPP: the goal is to learn an embedding mapping from sequences to the hyperbolic space while preserving backbone tree distances.

In this paper, we introduce the Hyperbolic Deep learning Enabled Phylogenetic Placement (H-DEPP) framework to find low-dimensional hyperbolic embeddings for gene sequences while preserving their distances in the backbone tree (Figure 1). Compared to the Euclidean approach [4], the low-dimensional hyperbolic embeddings lead to a neural network with fewer parameters, improved performance in estimating the phylogenetic distances for both reference and query organisms, and improved computational complexity of the training. We conclude by evaluating the performance of H-DEPP for placing new sequences on phylogenetic trees and updating the existing trees to include new species, testing both on simulated and real biological data.

## 2. Materials and Methods

We start with a formal presentation of our problem. We then briefly discuss limitations of Euclidean embeddings and move on to specific mathematical properties of hyperbolic spaces that are relevant to our work. Next, we introduce the design of the H-DEPP framework that incorporates hyperbolic embeddings in DEPP. We end by describing the design of our experiments evaluating H-DEPP.

### 2.1. Problem Statement

Let *T* be a weighted tree with leaves $V=\{v_{1},\ldots ,v_{N}\}$ and let $\left[N\right]\overset{\mathrm{def}.}{=}\left\{1,\ldots ,N\right\}$. We define $d_{T}:V\times V\to R^{+}$ to be the tree distance function; i.e., it gives the path length distance between vertex pairs in *T*. Let $s_{1},\ldots ,s_{N}\in S={\left\{A,C,T,G,-\right\}}^{L}$ be a set of aligned gene sequences (of length *L*) such that the sequence $s_{n}$ corresponds to $v_{n}\in V$ on the backbone tree, for all n∈[N]. An ideal embedding function $\phi^{*}$ maps the gene sequences to a metric space (M,d) while preserving the metric information of *T*; i.e.,

(1) $$\mathrm{for}\;\mathrm{all}\;i,j\in \left[N\right]:d\left(\phi^{*}\left(s_{i}\right),\phi^{*}\left(s_{j}\right)\right)=d_{T}\left(v_{i},v_{j}\right).$$

When the equality does not hold, we say that the embedding has distortion. For the novel sequence *s* from species v∉V, already aligned to the reference sequences, the distance between $\phi^{*}\left(s\right)$ and $\phi^{*}\left(s_{i}\right)\in M$ matches with the distance between *v* and $v_{i}$ if *v* is placed correctly on the backbone tree. When the given backbone tree *T* is a species tree and sequences are a set of single-gene sequence data, we call the problem discordant phylogenetic placement; and the goal is to the best approximate $\phi^{*}$ in (1).

### 2.2. Euclidean Embedding and Its Limitation

A perfect Euclidean embedding is a set of points $x_{1},\ldots ,x_{N}\in R^{N-1}$ such that

$$\mathrm{for}\;\mathrm{all}\;i,j\in \left[N\right]:d_{T}\left(v_{i},v_{j}\right)={∥x_{i}-x_{j}∥}_{2}^{2},$$

where ${∥\cdot ∥}_{2}$ denotes the $\ell_{2}$ norm.

Low-distortion embedding of trees requires a high-dimensional Euclidean space. Let *T* be a perfectly height-balanced binary tree with unit edge weights. In this tree with diameter 2r, the distance between the two closest leaves is 2, and for $2^{r}$ leaves, the distance of each leaf to the root is *r*. The low-distortion embedding of this tree in a *d*-dimensional Euclidean space maps each leaf inside the ball of diameter 2r, whose volume is $O(r^{d})$. As we increase the height (*r*) of the tree, the embedded leaves must collapse onto each other since the average volume associated with each one vanishes to zero, i.e, $\lim_{r\to \infty }r^{d}2^{-r}\to 0$. In addition, in a Euclidean ball of radius *r*, there has to be $O(2^{r}r^{-d})$ embedded leaves with underestimated distance (≤2). Moreover, Linial et al. [16] consider the problem of embedding a tree *T* with *N* vertices such that

$$\mathrm{for}\;\mathrm{all}\;\mathrm{distinct}\;i,j\in \left[N\right]:c^{-1}\leq \frac{∥\phi \left(v_{i}\right)-\phi \left(v_{j}\right){∥}_{2}}{d_{T}\left(v_{i},v_{j}\right)}\leq 1,$$

where ϕ maps each vertex to a point in $R^{d}$ and *c* is the distortion rate. They prove that the isometric embedding (c=1) of an arbitrary tree requires a Euclidean space with dimension d=O(log(N)), which grows with the size of the tree.

### 2.3. Embedding in Hyperbolic Spaces

Spherical and hyperbolic spaces are constant curvature spaces with positive and negative curvatures, respectively. We are interested in hyperbolic spaces because they are suitable for embedding vertices of metric trees [14]. A strong result by Sarkar [17] shows that trees can be embedded with arbitrarily low distortion into a hyperbolic space with only two dimensions. In hyperbolic spaces, the Riemannian metric defines lines (geodesics), distances, and volumes (Figure 1a) in ways that are different from Euclidean spaces and permit better embeddings of trees. For example, the volume of a *d*-dimensional hyperbolic ball of radius *r* grows exponentially with its radius, i.e., $O(e^{(d-1)r}r^{d})$, in contrast to the polynomial growth of the Euclidean ball. As Ganea et al. [11] point out, this exponential growth enables hyperbolic spaces to embed any weighted tree while preserving its metric with high fidelity.

#### 2.3.1. Hyperbolic Spaces in Machine Learning

The machine learning community has recently focused on hyperbolic spaces due to their improved embedding capabilities over Euclidean spaces to represent pairwise measurements such as distances, similarities, and hierarchies [9,10,11]. Several authors have recently adopted neural networks to learn representations in hyperbolic spaces in a wide variety of problems, e.g., classification, regression, detection, manifold learning, and high-dimensional distribution approximation. Hyperbolic neural networks have been developed to combine the representational power of hyperbolic geometry with the feature extraction capabilities of neural networks [12,13]. The main challenge in designing hyperbolic neural networks is performing hyperbolic arithmetic operations and deriving stable back-propagation formulas [12,13,15]. Learning in non-Euclidean spaces is quickly advancing with works such as constant curvature graph convolutional networks [18,19], hyperbolic graph neural networks [20,21], mixed-curvature variational autoencoders [22], and hyperbolic attention networks [23] to name a few.

Related to tree embedding, Matsumoto et al. [24] propose a modified version of the hyperbolic distance function to gain distance additivity for incident branches. They evaluate the performance of their method on embedding phylogenetic trees and integrating embeddings of different trees. They report numerical results on small trees (with only ≈100 leaves), and it is not clear how this modified distance function performs in representing additive distances of large trees. Corso et al. [25] propose a framework to embed sequences in geometric spaces, namely hyperbolic spaces, to tackle edit distance approximation, hierarchical clustering, and multiple sequence alignment problems, which are all important in bioinformatics. Their extensive numerical experiments show that (data-dependant) embedding methods outperform related (data-independent) classical approaches in terms of accuracy and inference speed. This work shows how using appropriate embedding space for biological measurements can improve the accuracy of relevant algorithms.

#### 2.3.2. Models of Hyperbolic Spaces

There are several isometric models of hyperbolic space, i.e., they are equivalent to each other in their embedding capabilities. Here we review *Poincaré ball* and *’Loid model*.

We denote the *d*-dimensional Poincaré ball of curvature C<0 as $I_{C}^{d}=\{x\in R^{d}:\sqrt{-C}{∥x∥}_{2}\leq 1\}$. The distance between two points is given by:

(2) $$\mathrm{for}\;\mathrm{all}\;x,y\in I_{C}^{d}:d\left(x,y\right)=\frac{1}{\sqrt{-C}}\mathrm{acosh}(1-2\frac{{C∥x-y∥}_{2}^{2}}{{(1+C∥x∥}^{2})(1+{C∥y∥}^{2})}).$$

We denote the *d*-dimensional ’Loid model with curvature C<0 as $L_{C}^{d}=\left\{x\in R^{d+1}:x^{⊤}Hx=C^{-1},x_{1}>0\right\}$, where $H=\mathrm{diag}\left(-1,1,1,\ldots ,1\right)\in R^{\left(d+1)\times (d+1\right)}$ and $x_{1}$ is the first element of vector *x*. We may represent the points in $L_{C}^{d}$ with *d*-dimensional free parameters as:

$$\mathrm{for}\;\mathrm{all}\;\;x^{\prime }\in R^{d}:x=\left[\begin{array}{c} \sqrt{-C^{-1}+{∥x^{\prime }∥}_{2}^{2}}\\ x^{\prime } \end{array}\right]\in L_{C}^{d}.$$

The distance between $x,y\in L_{C}^{d}$ is $d\left(x,y\right)=\frac{1}{\sqrt{-C}}\mathrm{acosh}\left(Cx^{⊤}Hy\right)$.

The unique properties of the two models can make each attractive in various applications. The Poincaré model facilitates a conformal (angle-preserving) visualization of the embedded points in a compact subset of $R^{d}$, whereas the domain of the ’Loid model is an unbounded subset of $R^{d+1}$. The ’Loid model provides a more stable distance function as Poincaré distance function becomes singular at its domain’s boundary, i.e., ${∥x∥}_{2}\to 1$. Therefore, the ’Loid model is more suitable for performing optimization tasks reliably [14].

#### 2.3.3. Hyperbolic Embedding Functions

In the Poincaré model, the one-to-one exponential map $\exp_{0}:R^{d}\to I_{C}^{d}$ is defined as:

(3) $$\mathrm{for}\;\mathrm{all}\;v\in R^{d}:\exp_{0}\left(v\right)=\frac{\tanh(\sqrt{-C}∥v{∥}_{2})}{\sqrt{-C}{∥v∥}_{2}}v\in I_{C}^{d}.$$

Similarly, let $e_{1}$ be the first standard basis for $R^{d+1}$ and $p_{\circ }\overset{\mathrm{def}.}{=}\frac{1}{\sqrt{-C}}e_{1}$. Then, in the ’Loid model, the exponential map at $p_{\circ }$ is defined as follows:

(4) $$\mathrm{for}\;\mathrm{all}\;v\in R^{d}:\exp_{p_{\circ }}\left(\left[\begin{array}{c} 0\\ v \end{array}\right]\right)=\cosh(\sqrt{-C}{∥v∥}_{2})p_{\circ }+\frac{\sinh(\sqrt{-C}∥v{∥}_{2})}{{∥v∥}_{2}}\left[\begin{array}{c} 0\\ v \end{array}\right].$$

The exponential map provides a way to map between Euclidean and hyperbolic spaces. Therefore, we may use the exponential map to convert a Euclidean embedding function to a hyperbolic one. In other words, if $\phi_{E}$ maps sequences to a Euclidean space, then $\phi_{H}\overset{\mathrm{def}.}{=}\exp\circ \phi_{E}$ maps the sequences into the related hyperbolic space.

### 2.4. H-Depp Design

The most obvious change needed to DEPP to adopt it to hyperbolic spaces is the loss function, which we discuss first. Then, we explore alternative ways to design neural networks that operate in the hyperbolic space. We end by pointing out a host of more subtle issues that arise when dealing with hyperbolic geometry.

#### 2.4.1. Loss Function

We want to find an embedding function that ensures the metric information in the backbone tree is best preserved in a hyperbolic space ($H^{d}$) — for the reasons outlined earlier. The empirically optimal embedding function is the minimizer of the cost function; i.e.,

(5) $${\hat{\phi }}_{N}=\underset{\phi \in \Phi }{arg min}E_{N}\left[{\left(\frac{d(\phi \left(s_{i}\right),\phi \left(s_{j}\right))}{d_{T}\left(v_{i},v_{j}\right)}-1\right)}^{2}\right],$$

where $E_{N}\left[\cdot \right]$ computes the empirical mean (average) over distinct indices, ${\left\{s_{n}\right\}}_{n\in [N]}$ are training sequences in S, Φ is the set of functions from S to $H^{d}$ represented by neural networks. The cost function (5) is comparable to the weighted least squares of Fitch and Margoliash [26], with the only difference being that, here, the weights are squared tree distances as opposed to sequence distances. The cost function (5) aims to minimize a multiplicative notion of embedding error as it operates on distance ratios.

The learned function ${\hat{\phi }}_{N}$ maps query sequences to a low-dimensional space where we can compute their evolutionary distances to the backbone species; i.e., $d({\hat{\phi }}_{N}\left(s\right),{\hat{\phi }}_{N}\left(s_{n}\right))$ for n∈[N], where *s* is a query gene sequence. If the model generalizes to unseen data, these estimated distances match with the tree distances for the true placement of the query. Then, we can use a distance-based placement method, such as APPLES-2 [6], to place the query on the backbone tree. The accuracy of new placements depends on the quality of estimated distances, i.e., the generalization error of the neural network.

#### 2.4.2. Neural Network Models

We use the same CNN model in DEPP [4], which uses one-hot encoding to represent sequences ($\frac{1}{4}$ for gaps) and three linear convolutional layers, with kernel sizes 1, 5, and 5, and a feed-forward from second to the third layer to make the latter a residual block, and a fully-connected layer with *d*-dimensional outputs. We study two alternative approaches to make a hyperbolic network:

1. *Exponential Maps:* We use a Euclidean neural network followed by the exponential map of the Poincaré ball or the ’Loid model shown in (3) and (4) and detailed in Section 2.3. We use the following cost function:
  $$\mathrm{cost}\left(\phi_{H},s\right)=E_{N}\left[{\left(s\frac{d(\phi_{H}\left(s_{i}\right),\phi_{H}\left(s_{j}\right))}{d_{T}\left(v_{i},v_{j}\right)}-1\right)}^{2}\right],$$
  where $\phi_{H}=\exp\circ \phi_{E}$, $\phi_{E}$ is the Euclidean neural network, exp(·) is the exponential map for the appropriate model of hyperbolic space with curvature −1 (i.e., (3) or (4)), and *s* is a scale factor further discussed in Section 2.4.3.
2. *HNN++:* We add one hyperbolic layer designed by [13] to the model used in DEPP. This all-by-all layer performs hyperbolic matrix multiplication; its inputs are the output of the previous Euclidean layer transformed by the exponential map, which outputs hyperbolic points used in the cost function (5). Finally, we perform parameter optimization using hyperbolic back-propagation [13].

#### 2.4.3. Training: Challenges and Solutions

We next discuss the practical issues in training and optimization in hyperbolic spaces, most of which are due to specific properties of hyperbolic spaces. The first issue is that the curvature parameter appears inside and outside the acosh(·) function and affects the domain of the Poincaré model; see (2). For simplicity, here, let us adopt the following notation:

(6) $$\mathrm{for}\;\mathrm{all}\;x\in I_{C}^{d}:x^{\prime }\overset{\mathrm{def}.}{=}\sqrt{-C}x\in I_{-1}^{d}.$$

One can show that $d\left(x_{1},x_{2}\right)=\frac{1}{\sqrt{-C}}d\left(x_{1}^{\prime },x_{2}^{\prime }\right)$, where we abuse the same notation d(·,·) to denote the appropriate distance function in each space; i.e.,

(7) $$\mathrm{for}\;\mathrm{all}\;x_{1},x_{2}\in I_{C}^{d}:d\left(x_{1},x_{2}\right)=s\;.\;\mathrm{acosh}\left(1+2\frac{∥x_{1}^{\prime }-x_{2}^{\prime }{∥}_{2}^{2}}{(1-∥x_{1}^{\prime }{∥}_{2}^{2})(1-∥x_{2}^{\prime }{∥}_{2}^{2})}\right),$$

where $x_{1}^{\prime },x_{2}^{\prime }\in \{x:R^{d}{:∥x∥}_{2}\leq 1\}$ and $s=\frac{1}{\sqrt{-C}}$. In the new distance function (7), the scale parameter *s* uniformly scales all pairwise distances. With the normalization transformation in (6), we make the domain of the new distance function invariant of the curvature parameter; see (7).

We may parameterize the cost function (5) as follows:

$$\mathrm{cost}\left(\phi ,s\right)=E_{N}\left[{\left(s\frac{d(\phi \left(s_{i}\right),\phi \left(s_{j}\right))}{d_{T}\left(v_{i},v_{j}\right)}-1\right)}^{2}\right],$$

where ϕ maps sequences to a hyperbolic space with curvature −1. For a fixed ϕ (neural network), cost(ϕ,s) is a convex function of *s* and has the following global minima:

(8) $$s_{\phi }^{*}=\left(E_{N}\left[\frac{d(\phi \left(s_{i}\right),\phi \left(s_{j}\right))}{d_{T}\left(v_{i},v_{j}\right)}\right]\right){\left(E_{N}\left[\frac{d^{2}\left(\phi \left(s_{i}\right),\phi \left(s_{j}\right)\right)}{d_{T}^{2}\left(v_{i},v_{j}\right)}\right]\right)}^{-1}.$$

The goal is to optimize the neural network and the scale parameter jointly. Updates according to (8) scale all pairwise distances and challenge the training of the neural network. We alternatively optimize the parameters of the neural network and the scale parameter using $s_{k+1}=s_{k}+\alpha_{k}\left(s_{\phi_{k}}^{*}-s_{k}\right),$ where $\phi_{k}$, $s_{k}$, and $\alpha_{k}$ are the neural network, scale, and the learning rate parameters at iteration *k*.

A second issue is that bit precision can impact the embedding accuracy. In a *d*-dimensional Poincaré ball, the distance between two points is given by,

$$d\left(x_{1},x_{2}\right)=\mathrm{acosh}\left(1+2\frac{∥x_{1}-x_{2}{∥}_{2}^{2}}{(1-∥x_{1}{∥}_{2}^{2})(1-∥x_{2}{∥}_{2}^{2})}\right),$$

where $x_{1},x_{2}\in \{x\in R^{d}{:∥x∥}_{2}<1\}$. Poincaré ball has a compact domain in $\ell_{2}$ sense. Points that are close to the boundary can have large distances. Suppose $∥x_{1}-x_{2}{∥}_{2}=\varepsilon$ where ε>0. For a diminishing value of ε, we can choose a large enough $\ell_{2}$ norm for each point to make $d(x_{1},x_{2})$ arbitrarily grow with $\varepsilon^{-1}$. For example, if $∥x_{1}{∥}_{2}={∥x_{2}∥}_{2}=({1-\frac{\sqrt{2}\varepsilon }{\sqrt{\cosh(\varepsilon^{-1})-1}})}^{\frac{1}{2}}$, then we have $d\left(x_{1},x_{2}\right)=\varepsilon^{-1}$. Therefore, distant points in a hyperbolic space can have similar vector representations in the $\ell_{2}$ sense.

Let $∥x_{n}{∥}_{2}^{2}\leq 1-10^{-p}$ for all points n∈[N] and a value of p∈N. Then,

$$d_{\max}\overset{\mathrm{def}.}{=}\max_{i,j\in [N]}d\left(x_{i},x_{j}\right)\leq 2\;.\;\mathrm{acosh}(1+2\frac{1-10^{-p}}{10^{-p}})\approx 4\log\left(2\right)+2\log\left(10\right)p\;.$$

Thus, the maximum hyperbolic distance between these points, $d_{\max}$, grows almost linearly with *p*; see Table 1. If the measured data have a large enough diameter ($d_{\max}\geq 100$), then the embedded tree vertices will be very close to the boundary, i.e., $∥x_{n}{∥}_{2}\to 1$, for any reasonable bit precision. Hence, an ε-inaccurate embedding will result in large embedding errors and computational complexities related to the singularity of the distance function on the boundary points, i.e., ${∥x∥}_{2}=1$. To remedy this issue, we normalize the tree distances to be at most 1, and the normalizing factor is absorbed in the scale factor in (7).

**Figure 2:**
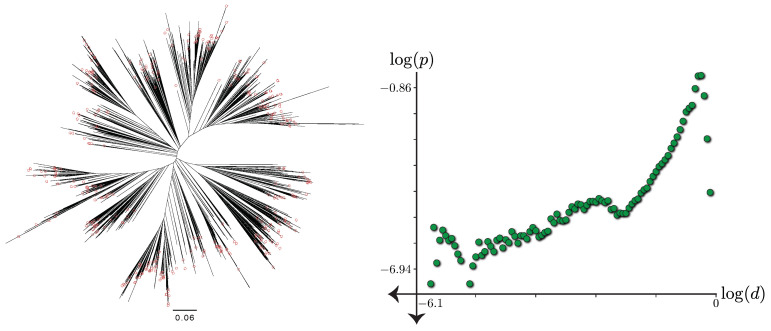
**Left**: The 7797-taxon reference tree adopted from Zhu et al. [7] restricted to sequences with 16S available with normalized lengths and query taxa noted. **Right**: The log-log scatter plot (base 10) for the histogram of pairwise tree distances *d* versus relative frequencies *p*.

A final issue is heterogeneous distances in reference trees. For example, on our biological dataset (discussed below), normalized tree distances vary by six orders of magnitude from 1 to $10^{-6.1}$ (Figure 2). Any gradient-based training method has to adaptively change the learning rate to estimate a point set whose wide range of distances are all accurate. Therefore, we propose to decrease the learning rate exponentially to cover a large range of erroneous distances. We let the learning rate at iteration *k* be $\alpha_{k}=\alpha_{0}p^{-\lfloor \frac{k}{K}\rfloor }$ where $\alpha_{0}$ is the initial learning rate, K=10 is the number of epochs with fixed learning rate, and p=0.95 is a decay factor.

### 2.5. Experimental Evaluation Setup

We start by describing a simulated and a biological dataset used in our analyses. We then detail our experimental procedure, evaluation criteria and the methods compared.

#### 2.5.1. Datasets

*Simulated dataset:* We use a published simulated dataset [27] where gene trees and species trees are discordant due to incomplete lineage sorting (ILS). We study the lowest and highest discordance models (both with a speciation rate of $10^{-6}$) from this dataset. Each model condition consists of 50 replicates, each with its own 201-taxon species tree. We select the first simulated gene (among 1000) without any identical sequences. We skip a replicate if none of its genes has all-unique alignments, a situation that happened for seven and three replicates in high- and low-discordance conditions. On average, the Robinson and Foulds [28] (RF) distance between the gene trees and species trees in the high- and low-discordance datasets are 0.69 and 0.21, respectively. Since sequences in this simulated dataset are gap-free, there is no need for alignment. As the backbone tree, we use the true species tree whose branch lengths are re-estimated using a concatenation of 32 genes, each with 500 randomly selected sites.

*WoL real data:* In 2019, Zhu et al. [7] published a dataset named Web-of-life (WoL). The dataset consists of 10,575 species and 281 marker genes. We use the available ASTRAL [27] tree that they provided with branch lengths recalculated using maximum likelihood (ML) from concatenated genes. We evaluate our method on the 16S gene in addition to another 10 marker genes selected among the 30 marker genes studied by Jiang et al. [4]. We select 10 out of 30 total marker genes to save computational resources. We first proceed by sorting genes according to their quartet distance to the species tree; we then select the first gene from every three genes in the sorted list, i.e., 1st, 4th, 7th, …, and 28th genes.

#### 2.5.2. Evaluation Procedure

We compare methods based on the accuracy of distances and trees. We measure the distortion of distances by calculating the squared error (SE) of embedding distances to the tree distances. We report both standard SE and SE weighted by the inverse of squared tree distances, which can be interpreted as the percentage by which the estimated distances deviate from true distances. Moreover, we note that while most distances are close to the true values, for a small number of outlier pairs, the weighted distortion is very high. Thus, we also examine the number of outliers in each distance matrix. Outliers are defined as distances whose weighted SE is larger than 100. Moreover, to avoid skewing the cost function due to outliers, we report the median of weighted SE.

To measure placement error, we randomly choose 5% species as queries and use the rest as the backbone. During testing, each query is placed onto the backbone tree independently. We measure the accuracy by counting the number of branches between the query position on the placement tree and the reference tree. We evaluate four methods.

- *Euclidean vs. Hyperbolic DEPP (E-DEPP vs. H-DEPP).* We use H-DEPP and E-DEPP models, trained on backbone data, to compute the distances between queries and backbone species. We then use the distance matrix as the input of APPLES-2 to find the placement of queries with parameter -b 5, which limits the placement to the five smallest distances. We use 128 dimensions by default.
- *APPLES2+Jukes-Cantor (JC).* APPLES-2 uses the Jukes and Cantor [29] (JC) model to compute sequence distances by default because Balaban et al. [6] found no evidence of improvement in placement accuracy by using more complex models. We use RAxML-ng to re-estimate the branch lengths of the backbone trees (JC model).
- *EPA-ng.* EPA-ng is a maximum likelihood placement method [30]. In our experiments, the backbone tree is inferred using RAxML-ng under the GTR+Γ model [31].

We also measure the error in updating trees. For simulated data, we use the same sets of genomes as query as those used in the placement task. For WoL data, we instead select queries in a clade-based approach in two replicates. We choose 100 clades with five to ten species from the reference tree and remove all these clades from the tree and add them to a query set. We then infer a fully resolved tree using four methods.

- *E-DEPP+FastME and H-DEPP+FastME.* We use FastME [32] for tree inference with the distance matrix computed partially using H/E-DEPP. We build a distance matrix that includes the tree distance for each pair of backbone species and the embedding distance computed using the H/E-DEPP model trained on the backbone data for other pairs. We use this approach to nudge FastME to retain backbone relationships because it does not have a constrained search option.
- *Jukes-Cantor (JC)+FastME.* The pipeline for tree update using JC is similar to H/E-DEPP. We first compute distances between all pairs of species using the JC model and build a distance matrix similarly to the H/D-DEPP model. We then use the distance matrix as the input of FastME.
- *RAxML.* We run RAxML [33] setting the backbone tree as a constraint (-g) under the GTR+CAT model.

To measure the accuracy of the tree update, we calculate the normalized quartet distance and RF distance between the estimated tree and the reference ASTRAL tree with species restricted to the queries. For 16S data, each species may contain multiple copies. We sample one copy for each species and prune the other copies from the estimated tree. We repeat this process 100 times and report the distribution of quartet/RF distances. For the marker genes data, we also test using multiple genes as input. For k∈{1…10} genes, we randomly select ten sets of genes, with each set consisting *k* genes. To use k>1 genes, we concatenate their sequences and use them as the input to RAxML and JC. For H-DEPP and E-DEPP, we follow the approach of Jiang et al. [4]: we build a model for each gene separately and use the median of distances across genes for each pair.

## 3. Results

We first compare different models of H-DEPP on the WoL 16S dataset, then compare H-DEPP with E-DEPP on both simulated and real datasets as the number of dimensions, and hyper-parameters change, next we compare H-DEPP with ML placement methods, and end by examining H-DEPP as a method of updating a tree to obtain a fully resolved tree.

### 3.1. Comparison between H-DEPP Alternatives

**Figure 3:**
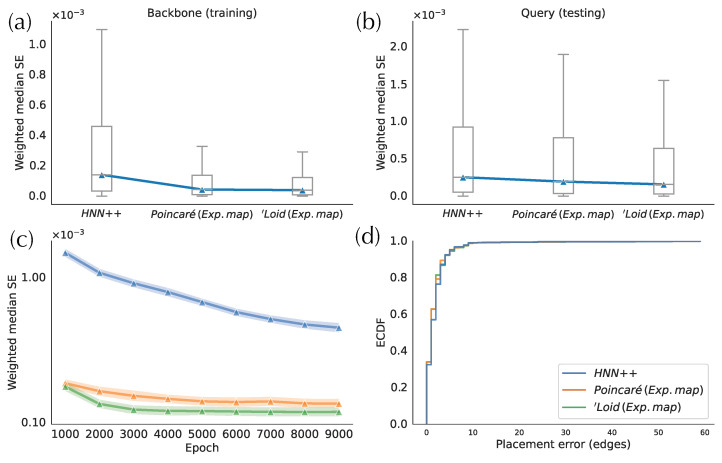
Comparison of different implementations of hyperbolic embeddings on WoL 16S data. (**a**) Mean square error (MSE) of embedding distances between backbone species. (**b**) MSE of embedding distances between query species and backbone species (**c**) Convergence of the models. (**d**) Empirical distribution function (ECDF) of the placement errors.

Compared to the two exponential map implementations, HNN++ has the worst distance distortion in both training and testing phases (Figure 3a,b). The HNN++ model, with one hyperbolic layer, has around two to threefold higher errors in testing and training sets, respectively. Moreover, HNN++ converges remarkably slower during training than the two alternatives (Figure 3c). For example, after 9000 training epochs, HNN++ still has a higher weighted median SE than the exponential map models trained for only 1000 epochs. This result indicates that the hyperbolic layer poses training challenges in accuracy and convergence rate. In comparison, in the exponential map approach, all operations in the training of the neural network are performed in the Euclidean space. The faster convergence rate may be due to avoiding complex arithmetic operations in the hyperbolic space, which are used in the hyperbolic layer to compute and back-propagate gradients of the cost function. Between the two models implemented with exponential maps, the ‘Loid model exhibits a smaller testing error and a faster training convergence compared to the Poincaré model (Figure 3c). While both models of hyperbolic space are mathematically equivalent (isometric), the divergent optimization efficiencies may stem from the limited operational precision and intricacies of the optimization algorithm.

Despite differences in the weighted median SE, the placement accuracy is similar across the three versions of H-DEPP (Figure 3d), a pattern that will be further discussed as we progress. Note also that the average placement error for all implementations is below two edges, which is already low for a backbone tree with more than 7000 species. since the ‘Loid model implemented with exponential map has the best performance overall, we use it as the default method in H-DEPP, including in the rest of the results.

### 3.2. H-Depp versus E-DEPP with Varying Number of Dimensions

We start by evaluating distance distortion before examining the impact on placement accuracy, which leads us to study the behavior of small distances.

#### 3.2.1. Distortion

**Figure 4:**
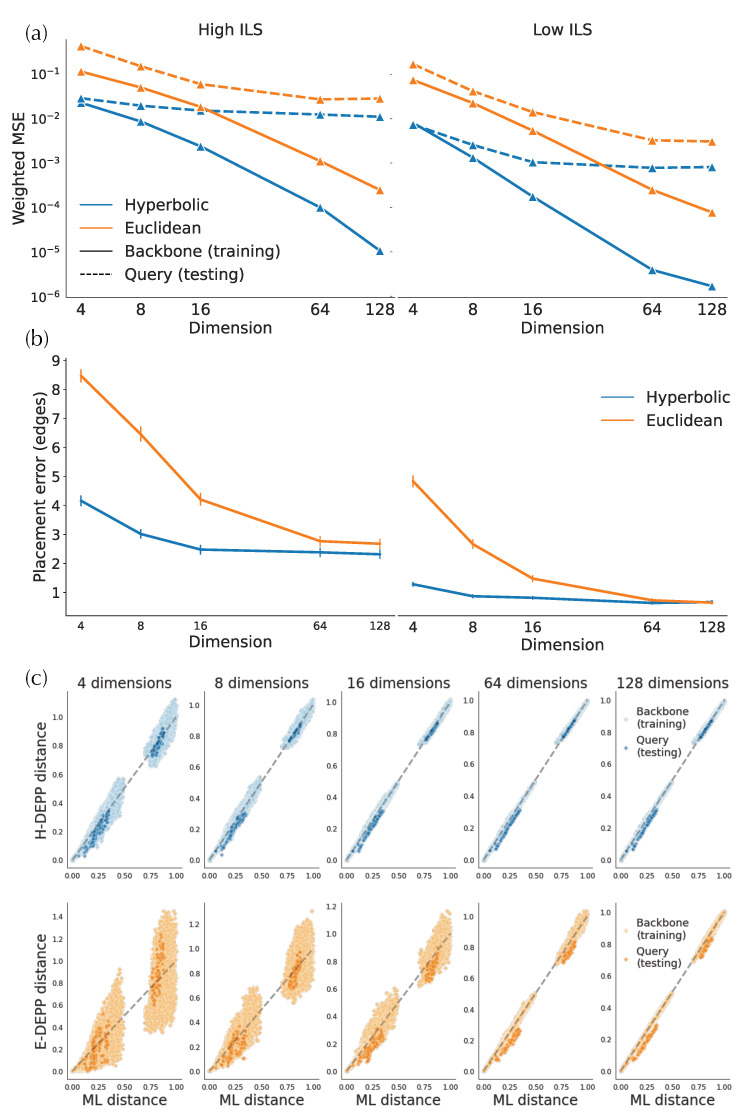
Hyperbolic and Euclidean embedding of simulated data. (**a**) Mean squared error (MSE) of embedding distances. (**b**) Placement error (**c**) True vs. estimated tree distance.

**Figure 5:**
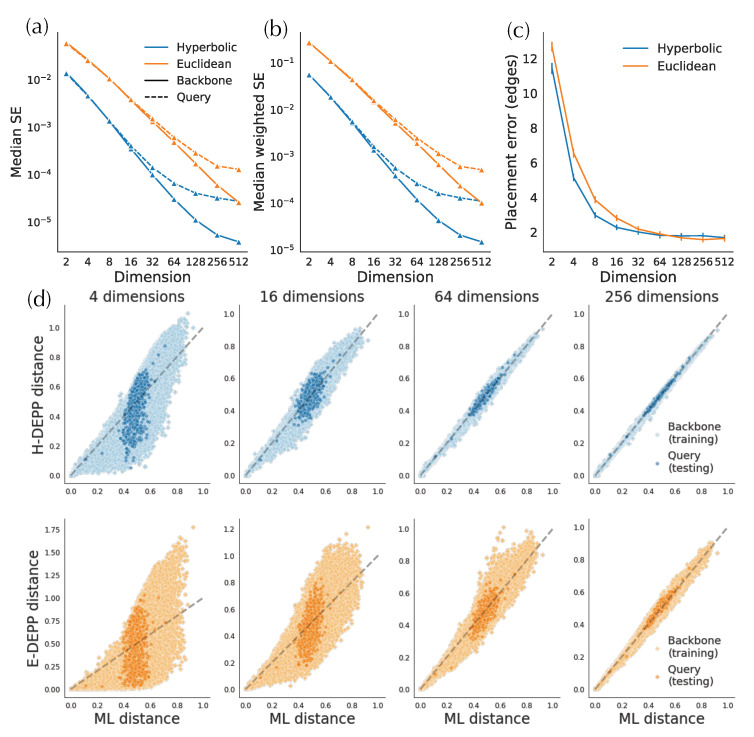
Hyperbolic and Euclidean embedding of WoL 16S data. (**a**) Median squared error of embedding distances. (**b**) Median squared error of embedding distances weighted by tree distances. (**c**) Placement errors (**d**) True vs. estimated tree distance.

**Figure A1:**
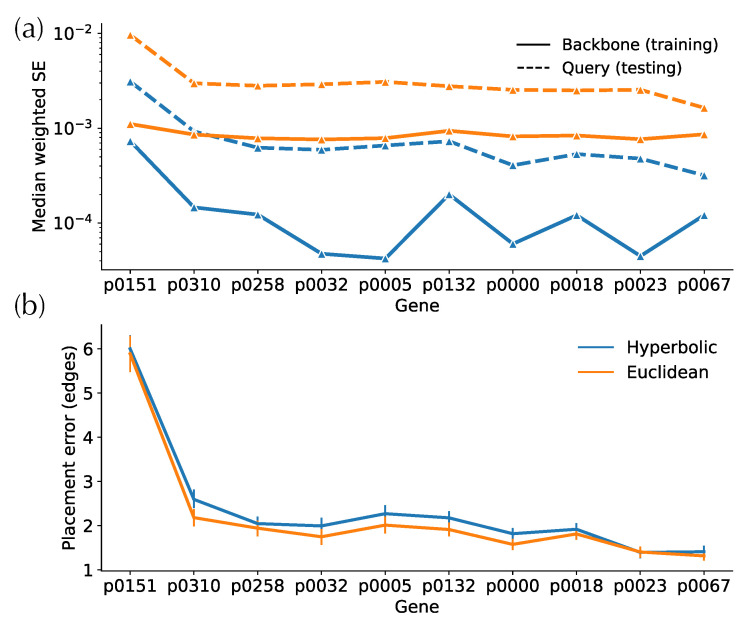
Comparison of Hyperbolic and Euclidean embeddings in WoL marker genes data. (**a**) Median square error of embedding distances. (**b**) Placement errors.

In simulated and biological data, hyperbolic embeddings have significantly lower distance distortion compared to the Euclidean embeddings as expected based on their theoretical advantages. On the simulated data, hyperbolic distances have one order of magnitude lower training error in both low- and high-discordance conditions, as well as one-half to one order lower testing error (Figure 4a). For example, on high-ILS simulated data, four hyperbolic dimensions are sufficient to get a query distance distortion comparable to that of 128-dimensional Euclidean embeddings in testing distances. In biological data, hyperbolic embeddings outperform the Euclidean counterparts with one-half to one order of magnitude in weighted MSE (Figure 5a,b and Figure A1a). For example, the training distortions in $H^{4}$ and $R^{16}$ are similar, as are the *query* distortions between $H^{32}$ and $R^{512}$. Finally, note that after a certain number of dimensions (e.g., 32 for WoL 16S data), the decrease in distance distortion of test samples slows down while training distortion continues to drop fast.

Examining the distances visually also shows that for low dimensions, hyperbolic embeddings lead to a significantly better distance estimation. For simulated data, only a four-dimensional hyperbolic space yields relatively low distortion distances (Figure 4c). However, in the larger WoL 16S dataset, despite the theoretical guarantees given infinite precision, the distortions are still at a considerable level, even with 16 dimensions (Figure 5d). Moreover, bias exists in both simulated and WoL 16S data with few dimensions and is more severe for queries than the backbone. For example, in simulated data, estimated query distances exhibit a noticeable bias compared to the true distances. The pattern is more obvious in Euclidean spaces as query distances of 16 (or more)-dimensional embeddings are significantly underestimated. Biases shrink or disappear with more dimensions.

#### 3.2.2. Placement Accuracy

A lowered distortion does not monotonically translate to a better placement performance. For the simulated data, the average placement error of H-DEPP is lower than Euclidean DEPP (E-DEPP) with low-dimensional embeddings (Figure 4b). With 128 dimensions, hyperbolic embedding outperforms the Euclidean counterpart on the high-ILS case, whereas they converge to similarly low levels of error (below one edge on average) in the low-ILS case. In the WoL 16S dataset, while hyperbolic embedding still greatly outperforms Euclidean embedding in low dimensions, it loses its advantages in higher dimensions (Figure 5c). When d≥128, both methods have reasonably low errors, and the performance of Euclidean embedding even slightly exceeds the hyperbolic one. In both datasets, as the dimensions increase, the rate of improvements in placement error diminishes. Note that with more dimensions, the reductions in testing distortion slow down. For example, on WoL 16S data, the unweighted mean squared error decreases from order of $10^{-2}$ to $10^{-3}$ as we go from d=2 to d=8; this reduction dramatically decreases the error from 12 edges to three. However, as we further reduce distortion (in orders $10^{-4}$ and $10^{-5}$) by adding more dimensions, the placement error stabilizes around its minimum. When we test other multiple marker genes of WoL data where we fix the number of dimensions to 128, Euclidean embedding shows a better placement than hyperbolic performance (Figure A1).

#### 3.2.3. Small Distances and Outliers

The lack of improvements in placement errors when distortion improves may seem counterintuitive across previous analyses (H-DEPP versions, more dimensions, and comparison to E-DEPP). However, APPLES-2 only uses a few small distances (the smallest five in our analyses) for finding the optimal placement. Thus, it can tolerate distortion among long distances beyond the natural tolerance for error [34,35] inherent in phylogenetic distance-based methods. As a result, APPLES-2 may be impacted more by distortion in small distances rather than large distances. Therefore, we further examine the accuracy of small distances (i.e., the smallest five distances for each species) versus the others.

**Figure 6:**
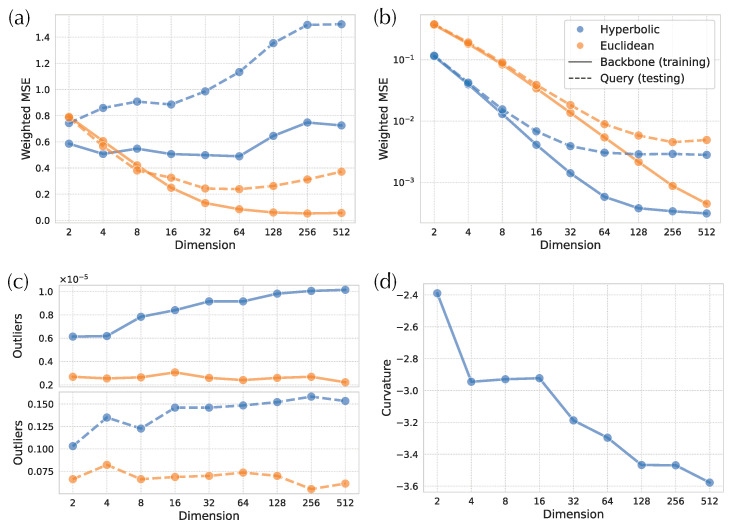
Examination of distortion on the 16S WoL dataset. (**a**,**b**) Weighted MSE for five smallest distances from each query (**a**) and all other distances (**b**). (**c**) Proportion of outliers among distances between pair of backbone species (**top**) and query and backbone species (**bottom**). Outliers: distances with weighted square error larger than 100; We remove outliers in either Euclidean distances or Hyperbolic distances for MSE calculation. (**d**) Curvature of Hyperbolic space.

Small and large distances have somewhat different patterns of distortion between Euclidean and hyperbolic embeddings (Figure 6a,b). Among larger distances, hyperbolic distortions are significantly smaller than the Euclidean ones, and the distortions of both hyperbolic and Euclidean distances reduce as the number of dimensions increases (Figure 6b). Although hyperbolic distortions decrease slower than the Euclidean ones for d≥64, they keep their advantage across all dimensions.

The distortions of small distances, however, exhibit a different pattern (Figure 6a). With very few dimensions, hyperbolic distances have a small advantage (only in training), but that advantage disappears quickly as the number of dimensions grows. In fact, for query distances, Euclidean embeddings can have one fourth of the distortion with enough dimensions. Moreover, testing distortion actually increases with high numbers of dimensions, pointing to potential overfitting.

There are outliers distances that have very high weighted distortions, and these tend to be among small distances (as expected since we weight the distances by the inverse of squared tree distances). While outliers are rare everywhere, Euclidean distances have far fewer outliers than hyperbolic in both training and testing data (Figure 6c). In the training data, the number of outliers tends to increase for hyperbolic but not Euclidean embeddings with the number of dimensions. The extra challenge in obtaining accurate small distances with hyperbolic methods might be due to the impact of curvature, a point we will revisit in discussions. On these data, the learned curvature parameter tends to decrease with the number of dimensions (Figure 6d).

### 3.3. Impact of Hyper-Parameters

DEPP’s stochastic optimization algorithm operates on randomly selected small batches of species (default size: 32). Some pairs may rarely be together in the same batch. To investigate whether this low probability can cause distortion in small distances and create outliers, we investigate the impact of batch size.

**Figure 7:**
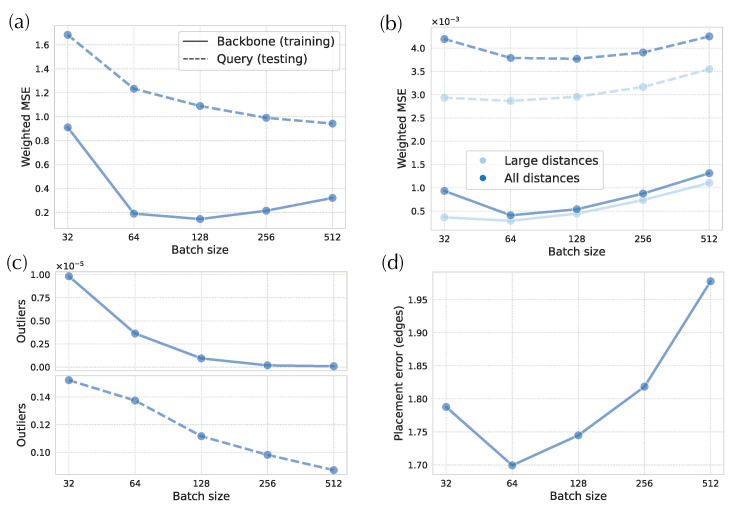
Impact of batch size on 16S data. (**a**) Weighted MSE for small distances. (**b**) Weighted MSE for large distances and all distances. (**c**) Proportion of outliers among distances between pair of backbone species (**top**) and distances between query and backbone species (**bottom**). (**d**) Placement errors.

As we increase the batch size, small-distance distortions significantly decrease at the testing time and reduce for batch size up to 128 at the time of training (Figure 7a). In the training data, the batch size 64 has a more than 70% reduction compared to the default 32. Inspecting the number of outliers shows a similar pattern (Figure 7c). For instance, if we use a batch size of 512, we can remove almost all outliers in the training phase and reduce them by one to twofold in the testing phase. However, the improvement in small distances and outliers comes at a high cost for distortions in large distances both in the training and testing phases for d>64 (Figure 7b). Both training and testing scores across *all* distances are minimized with batch sizes of 64 (not our default, 32), where small distances are improved, and large distances have not yet deteriorated. Incidentally, the optimal placement accuracy is also obtained with the batch size set to 64 (Figure 7d).

Since the models seem to struggle with learning and representing small distances (e.g., distances below $10^{-5}$), we examine a setting where we artificially remove the small distances from the tree by adding pseudo counts to the terminal branches. We test different values of pseudo counts (Table A1). With large enough pseudo counts (e.g., $10^{-3}$ or $10^{-4}$), distortions of small distances decrease, and the distortions of large distances slightly increase or remain comparable. In addition, there are far fewer outliers. However, comparison of distortion is not straightforward because errors are normalized by the square of the inverse of tree distances. When we examine the placement errors, which do provide a fair comparison (unlike the normalized MSE), none of the pseudo-count values result in better placement accuracy.

### 3.4. Placement Accuracy: Comparison to Alternatives

**Figure 8:**
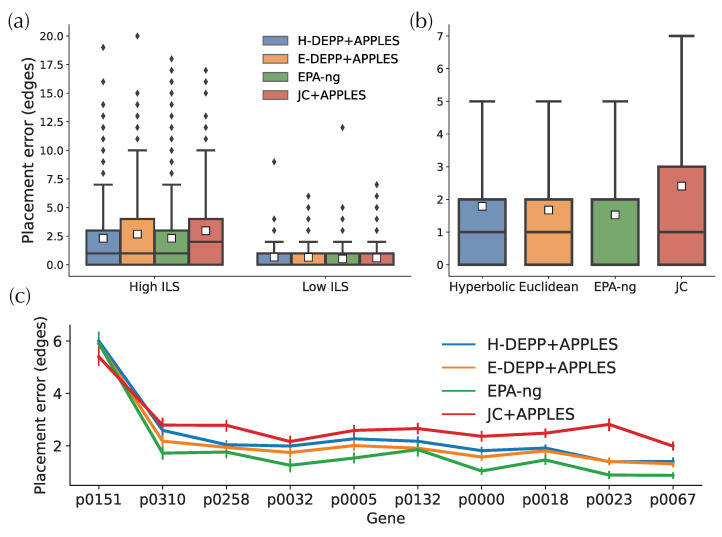
Phylogenetic placement for (**a**) simulated data (**b**) WoL 16S data (**c**) WoL marker genes data. The white squares in the boxplots represent the average placement errors.

Having established that H-DEPP does not consistently improve over E-DEPP in placement accuracy, we now compare it to other methods. In the high ILS simulated dataset (Figure 8a), H-DEPP has a lower average placement error and a shorter tail of error than JC+APPLES and is comparable to EPA-ng (both with 2.3 edges of error on average). In the low ILS case, where the placement tasks are less challenging, all four methods are low in placement errors with an average of ∼0.6 edges. On the WoL 16S data (Figure 8b), EPA-ng finds the correct placement 50% of times, compared to 40% for E-DEPP and 33% for H-DEPP. In terms of error, the performance of H-DEPP and E-DEPP is comparable with the same median error, and H-DEPP has a slightly higher average error (1.78 vs. 1.67 edges). The mean error of JC is significantly higher than the other alternatives (2.4 edges).

On the other WoL marker genes, EPA-ng continues to have the best performance (Figure 8c). Neither H-DEPP nor E-DEPP matches the accuracy of EPA-ng using 128 dimensions. Across all genes, the average error is 2.3, 2.1, 1.8, and 2.8 edges for H-DEPP, E-DEPP, EPA-ng, and JC, respectively. Interestingly, compared with EPA-ng, both H-DEPP and E-DEPP have fewer highly-erroneous outlier placements. For example, only 1.6% of all placements are at least 15 edges away from the optimal placements for H-DEPP compared to 2.6% for EPA-ng.

### 3.5. Tree Extension: Comparisons to Alternatives

**Figure 9:**
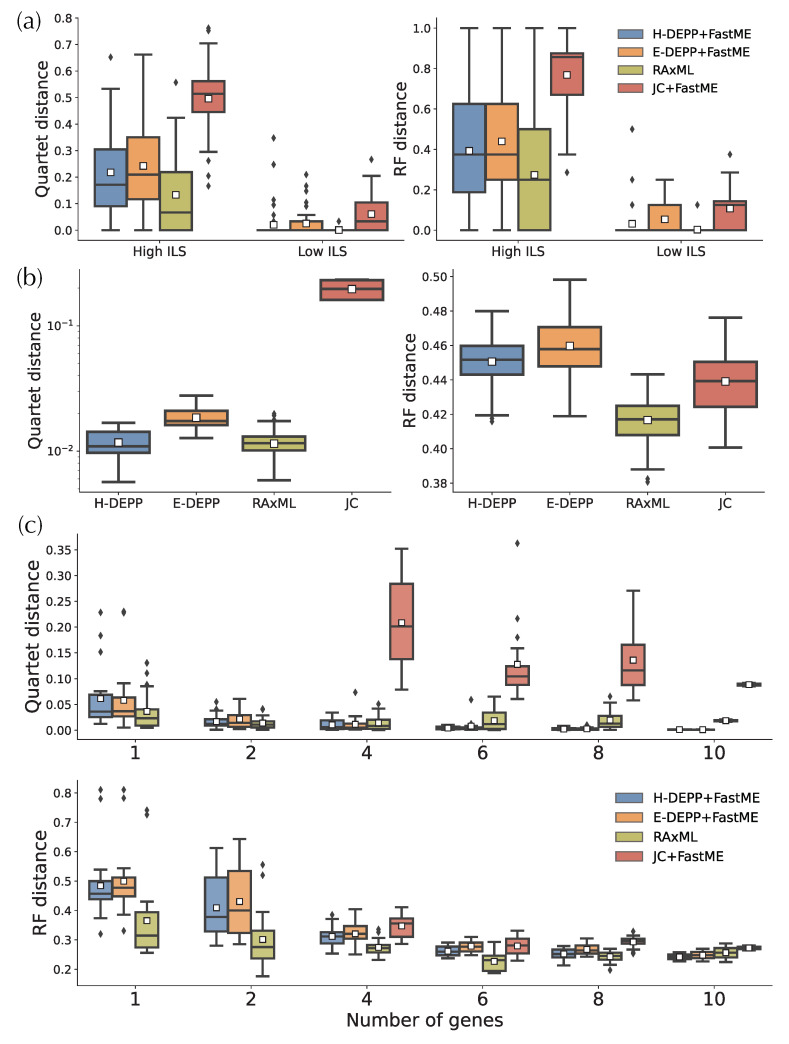
Tree update for (**a**) simulated data (**b**) WoL 16S data (**c**) WoL marker genes data. The white squares in the boxplots represent the average over all replicates.

When updating trees to get fully resolved trees, on the simulated data, H-DEPP has slightly lower average errors compared to E-DEPP but higher errors than RAxML (Figure 9a). In particular, in the low ILS condition, 83% of H-DEPP updated trees have zero error, compared to only 70% for E-DEPP. Trees updated using FastME with JC distances are far more erroneous compared to the other methods. Thus, the relatively high accuracy of DEPP+FastME is due to its improved distances, not the advantages of FastME. On the WoL data, we see similar patterns with the 16S gene (Figure 9b). H-DEPP has clear advantages over E-DEPP, and H-DEPP and RAxML are comparable when judged using quartet score (0.011 versus 0.012 on average). With the RF measure of error, although H-DEPP still outperforms E-DEPP, both methods are worse than RAxML and JC, and all methods have high error. RF is sensitive to rogue taxa, and the presence of paralogous 16S copies and HGT can create unstable leaves. So there may be several outlier leaves in H/E-DEPP trees that are very far away from their correct positions and skew the error measured by RF distances.

When we use one or two WoL marker genes, RAxML continues to exhibit advantages over the other alternatives (Figure 9c). For one or two genes, H-DEPP and E-DEPP share a similar median and average quartet distance, while the RF distances for H-DEPP is slightly lower than E-DEPP. Note that we cannot compute JC distances when the alignment contains pairs of species with non-overlapping non-gap sequences. Such situations occasionally happen when we have one or two genes. Since FastME fails when the input distance matrix contains missing data, we omit comparisons to JC for one or two genes.

**Figure A2:**
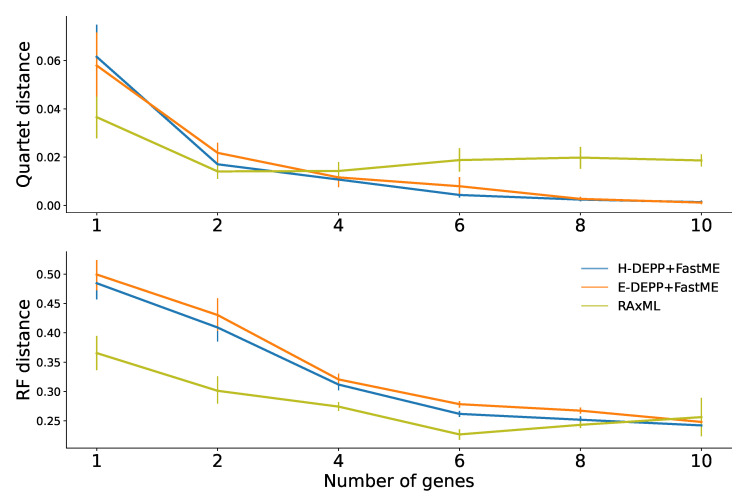
Errors of updated trees measured by (**top**) quartet distances; (**bottom**) RF distances; Results are from WoL marker genes data.

As the number of genes increases, patterns change (Figure 9c). The performance of H-DEPP and E-DEPP improves with increasing numbers of genes according to quartet and RF measures (Figure 9c and Figure A2). For RAxML, however, quartet and RF errors stabilize or even slightly increase beyond two or six genes, respectively. The error of H/E-DEPP is lower than RAxML when a handful of genes are available (four for quartet distances and 10 for RF distances). The improvements in quartet error become dramatic when the number of genes increases to 10: the mean error of H-DEPP is 0.0013 compared to 0.018 for RAxML.

## 4. Discussion

H-DEPP improved distances compared to E-DEPP on all the datasets. While our attempts at improving single-gene placement using hyperbolic embedding were not universally fruitful, we did improve the accuracy of updates (i.e., producing a fully resolved tree with new queries) going from E-DEPP to H-DEPP. Thus, the improvement in distance fidelity is not in vain. We next describe what can cause these diverging patterns.

### 4.1. Tree Updates and the Impact of Discordance

We observed that H-DEPP was better than RAxML in updating the reference ASTRAL tree using several genes. While updating a species tree using a concatenation of a few genes is a difficult task, H-DEPP, unlike RAxML, had remarkably low quartet errors (Figure 9). This was in contrast to the case with one gene where RAxML was comparable or better than H-DEPP. One explanation for the change in performance with more genes is that genes that are highly discordant with the species tree affect the performance of RAxML. H/E-DEPP is able to reduce the impact of outlier genes by using the median of the distances. To further examine this pattern, we examine the correlation between the quartet distance of the species tree to gene trees and the species tree error (Table 2). For H-DEPP and E-DEPP, species tree quartet error correlates significantly and strongly with the smallest two gene tree quartet distance values, whereas it has no correlation with the max gene tree quartet distance and a much weaker (albeit potentially significant) correlation with the mean. In contrast, RAxML correlates most strongly with the maximum quartet score, meaning that genes with high discordance with the species tree impact the performance of RAxML. Therefore, the better performance of H-DEPP can be attributed to better handling of discordance.

Practically, the strong performance of H-DEPP opens up the path for using deep learning to update species trees using a handful of genes. This problem, which is more ambitious than gene tree updating, can be very impactful if achieved with high accuracy and using few marker genes. In principle, existing species trees with many tens of thousands of species, e.g., [7,8] can be updated to include new species using just a few marker genes. Given the remarkably accurate trees obtained using as few as six genes using H-DEPP (Figure 9), we can perhaps update these large species trees using only a handful of genes.

### 4.2. Placement Accuracy and the Impact of Small Distances

Unlike updates, placement accuracy did not improve, leading us to a question: Why does placement accuracy fail to change with improved distance calculations? The reason is likely the reliance of APPLES-2 on the smallest distances for placement. Consistent with this explanation, we saw that while hyperbolic embeddings provided much lower distortion overall, they had higher distortion for small distances. They also had more cases of outlier pairs that have extremely high distortion. When we improved the accuracy of small distances and reduced outliers by increasing the batch size to 64 (without sacrificing long distances), we did see improvements in placement accuracy. Thus, small distances seem to matter more for placement. Accepting this explanation leads to the next question.

Why is hyperbolic embedding worse than Euclidean in estimating small distances, and why do small distances not improve with more dimensions? Computing accurate distances and distance gradients for two close points both near the boundary of the curved hyperbolic space require the calculation of fast-growing functions (Figure 1). Such operations can become inaccurate with limited precision floating point operations. The uncurved Euclidean spaces do not suffer a similar problem. To make things more complex, we also trained curvature, which tended to reduce with more dimensions (Figure 6d). Had the curvature not decreased, the embedded points would have had to collapse closer to the origin with more dimensions because the diameter is fixed. Instead, the algorithm learned to decrease the curvature so that values of each point along individual dimensions stayed larger, perhaps to improve the precision across all distances. However, the decreased curvature pushed the points close to the boundaries where small distances, specifically, are harder to fine-tune.

Another potential explanation is that perhaps our training is stuck in local optima and the local optima are particularly problematic for small distances. In particular, as we increase the number of dimensions, the optimization space changes, and it may include more local optima, leading to the lack of improvement in small distances with more dimensions (Figure 6a); also note that we kept the number of epochs of training fixed as we changed dimensions. Another factor contributing to convergence is that our stochastic gradient descent optimizer randomly groups species into batches in each epoch. Given a large number of species, there is a non-negligible probability of never sampling two pairs of close species in the same batch. For example, with the default batch size (32) on the WoL 16S dataset with 7800 taxa, two sister species have a ${\left(1-\frac{32}{7800}\right)}^{1000}=1.6$% chance of never appearing in the same batch in 1000 consecutive epochs. The accuracy of small distances can be expected to be more prone to a lack of joint sampling than large distances because distances of leaves far on the tree correlate with many other distances, whereas distances of species close on the tree (e.g., sisters) correlate with far fewer distances. Therefore, training for accurate small distances may be more dependent on having them in the same batch compared to the larger ones. Consistent with this idea, we saw that with increased batch size, the small distances improved while larger ones did not (Figure 7).

While there is evidence that smaller distances matter more, we also observed that larger distances cannot be fully sacrificed. Increasing batch size beyond 128 reduced placement accuracy compared to default 32 despite having better small distances at the expense of large distances (Figure 7). The decreased accuracy of large distances with increased batch size was surprising, and we do not have a strong explanation for it. Perhaps, by further emphasizing small distances (which contribute more to the loss function due to the weighting), gradients were less impacted by large distances, and hence, those were de-emphasized. Or perhaps, the models tried to fit the small distances for even challenging species (e.g., species with horizontal gene transfer) by sacrificing generalization over all the data. Regardless of the cause, small distances matter more, but larger ones also do matter.

### 4.3. Future Research

We saw that the choice of some hyper-parameters, such as batch sizes and learning rates, is consequential. In practice, the best choice of hyper-parameters is expected to be dataset-dependent. While it was not practical on our large number of datasets used for benchmarking, when used on real data, it would be best to choose the hyper-parameters in a two-step approach by using a validation set. Bilevel optimization techniques abound in the machine learning literature, e.g., [36,37,38] and can be adapted to facilitate this goal in future work.

Future work can test H-DEPP under more varied conditions. Most importantly, microbiome analyses often are based on fragmentary data, which were not tested here systematically. Moreover, in our tests, we used an alignment that was built based on queries and reference sequences, pruned down to include the backbone in training. While UPP aligns the vast majority of sequences independently and one by one, there is still room for some leakage of information (at least for the alignment) from testing to training because of the shared alignment. More broadly, we did not explore the impacts of alignment errors on the results, which may affect methods differently. Finally, we did not directly study the impact of the number of species. It is reasonable to expect that smaller trees can be trained better than larger trees, and therefore, a divide-and-conquer approach using an ensemble of models may further improve accuracy.

## 5. Conclusions

In this paper, we showed that hyperbolic geometry can improve the fidelity of distances computed using the deep learning framework DEPP from a reference dataset. Much fewer hyperbolic dimensions are enough to obtain the same level of distortions as the Euclidean space. This improvement in distances comes at no significant computational cost because the training and testing times of H-DEPP and E-DEPP are similar. In fact, by reducing the required number of dimensions, H-DEPP can reduce the computational cost. Despite the promising improvements in distances, the impact on placement accuracy was mixed. We observed improved accuracy in the high ILS simulated dataset, unchanged accuracy in many of the other datasets, and even reduced accuracy in some cases. We did, however, obtain improved accuracy for the more ambitious task of tree updates where a fully resolved tree is inferred using the existing tree for training the model. We further explored the reasons behind these patterns. Our proposed explanations collectively point to a complex set of factors contributing to the distortion, update, and placement accuracy.

We identified many challenges in using hyperbolic embeddings. Our solutions included training the curvature jointly with data, scaling distances to avoid low precision calculations, a dynamically adjusted learning rate, and using exponential maps instead of HNNs. All these techniques and observations may prove useful beyond phylogenetics, where hyperbolic neural networks are used increasingly. Similarly, further improvements in methods for training hyperbolic neural networks in the machine learning community can be adopted in H-DEPP for improved accuracy.

## Figures and Tables

**Table 1:** The maximum distance ($d_{\max}$) as a function of the bit precision (*p*).

*p*	2	4	6	8	10	12	14	16	18	20	22	24
$d_{\max}$	11.97	21.19	30.40	39.61	48.82	58.03	67.24	76.45	85.66	94.87	104.08	113.29

**Table 2:** Drives of errors in updating trees. We show the correlation between the errors of the estimated tree measured by quartet distance and $\bar{qd}$: mean of QD, $\bar{qd_{min}}$: mean of the minimum two values in QD, and $qd_{max}$: maximum value in QD, where QD is the set of quartet distances between the gene trees corresponding to each input gene and the species tree. We use Wald test on the linear least-square regression with the null hypothesis of zero slope. *r*: the Pearson correlation coefficient.

	$\bar{\mathrm{qd}}$		$\bar{{\mathrm{qd}}_{\min}}$		${\mathrm{qd}}_{\max}$	
	***p*** **-Value**	r	***p*** **-Value**	r	***p*** **-Value**	r
H-DEPP	0.012	0.29	$2.61\times 10^{-8}$	0.59	0.57	−0.06
E-DEPP	0.061	0.21	$1.14\times 10^{-4}$	0.43	0.48	−0.08
RAxML	0.474	0.08	0.86	−0.02	0.08	0.20

## Data Availability

Data for this paper are available from GitHub at https://github.com/yueyujiang/hyperbolic_results, accessed on 20 August 2022. The code is available at https://github.com/yueyujiang/hdepp, accessed on 20 August 2022.

## Appendix A. Extra Tables

**Table A1:** Impact of adding pseudo counts to terminal branch. MSE: MSE of full distance matrix; MSE (small): MSE for the smallest five distances for each species; MSE (large): MSE for all the distances except the small distances; upper: results for backbone species; bottom: results for query species.

**Pseudo Count**	**MSE**	**MSE (Small)**	**MSE (Large)**	**#Outliers**	
0	0.00093	0.74	0.00036	416	
0.001	0.00066	0.33	0.00041	170	
0.0001	0.00072	0.52	0.00033	320	
0.00001	0.00145	1.35	0.00042	450	
**Pseudo Count**	**MSE**	**MSE (Small)**	**MSE (Large)**	**#Outliers**	**Placement Error**
0	0.0041	1.68	0.00293	250	1.78
0.001	0.0037	0.99	0.00296	146	1.80
0.0001	0.0039	1.39	0.00292	237	1.78
0.00001	0.0042	1.77	0.00296	250	1.75

## Appendix C. Commands and Versions

### Appendix C.1. Software Version

- APPLES 2.0.0
- DEPP 0.2.2
- EPA-ng 0.3.8
- RAxML-ng 1.0.1
- RAxML 8.2.12
- UPP 4.3.10
- FastME 2.1.6.1

### Appendix C.2. Branch Length Reestimation

- raxml-ng –evaluate –msa $sequence_file
  –tree $tree –model JC –threads 2
  –blopt nr_safe

### Appendix C.3. Gene Alignment

Genes were aligned using UPP using the following commands for aligning the backbone sequences(first command), and aligning the novel queries to the existing backbone alignments (second command).

- run_upp.py -s $input_seq -B 2000 -M -1 -T 0.33 -A 200
- run_upp.py
  -s $query_seq
  -a $backbone_seq
  -t $backbone_tree
  -A 100 -d $outdir

### Appendix C.4. Placement

- APPLES+E-DEPP
  − Training
    ∗ Simulated dataset
      train_depp.py
      backbone_tree_file=$backbone_tree
      backbone_seq_file=$backbone_seq
      patience=5 lr=1e-4 embedding_size=128
    ∗ WoL dataset
      train_depp.py
      backbone_tree_file=$backbone_tree
      backbone_seq_file=$backbone_seq
      patience=5 lr=1e-4 embedding_size=128
  − Query time
    ∗ Calculating distance matrix
      · depp_distance.py
        query_seq_file=$query_seq
        backbone_seq_file=$backbone_seq
        model_path=$model_path
        outdir=$out_dir
    ∗ Placement using APPLES
      · run_apples.py
        -d $distance_file
        -t $backbone_tree
        -o -f 0 -b 5
- APPLES+H-DEPP
  − Training
    ∗ Simulated dataset
      train_depp.py
      backbone_tree_file=$backbone_tree
      backbone_seq_file=$backbone_seq
      weighted_method=’fm’
      patience=5 lr=1e-4 embedding_size=128
      c_epoch=6000 clr=0.005 distance_mode=’hyperbolic’
    ∗ WoL dataset
      train_depp.py
      backbone_tree_file=$backbone_tree
      backbone_seq_file=$backbone_seq
      weighted_method=’fm’
      patience=5 lr=1e-4 embedding_size=128
      c_epoch=6000 clr=0.005 distance_mode=’hyperbolic’
  − Query time
    ∗ Calculating distance matrix
      · depp_distance.py
        query_seq_file=$query_seq
        backbone_seq_file=$backbone_seq
        model_path=$model_path
        outdir=$out_dir
    ∗ Placement using APPLES
      · run_apples.py
        -d $distance_file
        -t $backbone_tree
        -o -f 0 -b 5
- APPLES+JC
  run_apples.py
  -s $backbone_tree
  -q $query_seq
  -t $backbone_tree
  -f 0 -b 5 -D
- EPA-ng
  − raxml-ng
    –evaluate
    –msa $backbone_seq
    –tree $backbone_tree
    –prefix info
    –model GTR+G+F
    –threads 2 –blopt nr_safe
  − epa-ng
    –ref-msa $backbone_seq
    –tree $backbone_tree
    –query $query_seq
    –model $GTR_info

### Appendix C.5. Tree Update

- FastME
  − Tree building
    ∗ fastme -i $input_dist -o $output_tree –nni -s -m B
  − JC distances
    ∗ fastme -i $input_seq -O $output_dist –dna=JC69 -c
- RAxML
  − raxmlHPC-PTHREADS
    -s $input_seq
    -w $output_dir
    -n run -p 12345
    -T 32 -m GTRCAT
    -g $backbone_tree
